# How frequently are predicted peptides actually recognized by CD8 cells?

**DOI:** 10.1007/s00262-016-1840-7

**Published:** 2016-04-23

**Authors:** Ioana Moldovan, Oleg Targoni, Wenji Zhang, Srividya Sundararaman, Paul V. Lehmann

**Affiliations:** Cellular Technology Ltd., 20521 Chagrin Blvd., Shaker Hts., OH 44122 USA

**Keywords:** Immune monitoring, Predicted antigenic peptides, Peptide pools, Tetramers, dextramers, multimers, CITIM 2015

## Abstract

Detection of antigen-specific CD8 cells frequently relies on the use of peptides that are predicted to bind to HLA Class I molecules or have been shown to induce immune responses. There is extensive knowledge on individual HLA alleles’ peptide-binding requirements, and immunogenic peptides for many antigens have been defined. The 32 individual peptides that comprise the CEF peptide pool represent such well-defined peptide determinants for Cytomegalo-, Epstein–barr-, and Influenza virus. We tested the accuracy of these peptide recognition predictions on 42 healthy human donors that have been high-resolution HLA-typed. According to the predictions, 241 recall responses should have been detected in these donors. Actual testing showed that 36 (15 %) of the predicted CD8 cell responses occurred in the high frequency range, 41 (17 %) in mid-frequencies, and 45 (19 %) were at the detection limit. In 119 instances (49 %), the predicted peptides were not targeted by CD8 cells detectably. The individual CEF peptides were recognized in an unpredicted fashion in 57 test cases. Moreover, the frequency of CD8 cells responding to a single peptide did not reflect on the number of CD8 cells targeting other determinants on the same antigen. Thus, reliance on one or a few predicted peptides provides a rather inaccurate assessment of antigen-specific CD8 cell immunity, strongly arguing for the use of peptide pools for immune monitoring.

## Introduction

CD8 cells recognize 9- to 10-amino-acid-long peptide fragments of protein antigens, including tumor antigens. Such antigenic peptides are displayed to the T cell receptors (TCR) of CD8 cells on MHC Class I molecules of antigen-presenting cells (APC). The antigenic peptides are bound in a dedicated peptide-binding groove of the Class I molecule with certain amino acids of the peptide chain attaching to the Class I molecule (MHC contact residues) while other residues are facing the TCR (TCR contact residues) [1]. There are over 100 MHC/HLA Class I alleles in the human population distributed over three loci of the human leukocyte antigen gene complex (HLA)) Of this sizable library of alleles, each human subject expresses up to six different HLA Class 1 molecules, constituting his/her unique HLA type [2].

Each allelic HLA molecule variant has a unique peptide-binding specificity dictated by differences in the amino acid sequence within the peptide-binding groove [3]. The expression of a certain HLA allele in any given individual will therefore predetermine what peptide segment(s) of an antigen can be presented to CD8 cells in that individual. CD8 cells in individuals with shared HLA Class I alleles can respond to the same peptide of a given protein antigen, but CD8 cells in individuals expressing different HLA alleles will respond to different peptides. Peptide recognition by CD8 cells is therefore highly variable among individuals of an outbred population, as variable as the HLA system itself, the latter primarily dictating the former. This becomes particularly important for understanding the variability of the anti-tumor T cell response in different individuals. It is thought that HLA polymorphism and polygenism and the resulting individualized peptide recognition have evolved to protect the species from mistakes of self-/non-self-discrimination by T cells ensuring that complications of infections or autoimmune diseases would affect only individuals with certain HLA alleles, but not endanger the entire population [4].

Since allelic HLA molecules dictate what peptides can be recognized by CD8 cells, considerable effort has been invested in the past two decades in understanding the rules of HLA-peptide binding. Peptide-binding motifs have been identified for most Class I alleles that permit us to predict peptide sequences of the antigen that are likely to bind to a given allele with high accuracy, and thus predict peptides that are potential antigenic determinants for CD8 cell recognition in individuals expressing that HLA allele [5–7]. The precision of these models has been extensively verified in vitro by HLA-peptide-binding studies.

Therefore, presently, we have a comprehensive knowledge base about peptides of antigens that can bind to the different HLA allelic molecules, and therefore we can identify peptides that can be presented on APC to CD8 cells in the context of an individual’s unique HLA type. However, the downstream consequences of antigen presentation are less known. From the many peptides predicted to bind to HLA molecules expressed in a host, often only few are actually recognized by CD8 cells. In a systematic study by Anthony et al., the CD8 cell response to the Core protein of Hepatitis C virus (a small antigen of 192 amino acids long) was studied [8]. Peptide prediction algorithms suggested that 43 nonamer peptides could bind to the HLA Class I alleles expressed in an individual. However, only two adjacent peptides (constituting a single determinant) were actually recognized by CD8 cells when all possible nonamers of 183 individual peptides that walked the sequence of the core antigen in steps of single amino acids were tested.

Presently it is unknown why CD8 cell recognition targets only few of the peptides on an antigen that can bind to HLA molecules expressed in a given individual. For many antigens, such immune dominant peptides—also called determinants—have been defined empirically, by studying the CD8 cell response itself. The 32 individual peptides that comprise the CEF peptide pool, that is widely used as a positive control for CD8 cell testing, represent such well-defined peptide determinants for Cytomegalo-, Epstein–barr, and Flu virus (Table 1) [9]. Each of these peptides has been verified to be recognized by CD8 cells, and the respective HLA allele used for antigen presentation has been defined (see references in [9]).

**Table 1:** List of CEF peptides and their restricting MHC Class I alleles as originally defined

CEF #	Virus	Antigen source	Epitope sequence	HLA Restriction
CEF-01	Influenza	PB1 (591–599)	VSDGGPNLY	A1
CEF-02	Influenza	NP(44–52)	CTELKLSDY	A1
CEF-03	Influenza	M1 (58–66)	GILGFVFTL	A2
CEF-04	Influenza	PA (46–54)	FMYSDFHFI	A2
CEF-05	EBV	LMP2A (426–434)	CLGGLLTMV	A2
CEF-06	EBV	BMLF1 (259–267)	GLCTLVAML	A2
CEF-07	HCMV	pp65 (495–503)	NLVPMVATV	A2
CEF-08	Influenza	NP (91–99)	KTGGPIYKR	Aw68
CEF-09	Influenza	NP (342–351)	RVLSFIKGTK	A3
CEF-10	Influenza	NP(265–273)	ILRGSVAHK	A3
CEF-11	EBV	BRLF1 (148–156)	RVRAYTYSK	A3
CEF-12	EBV	EBNA 3a (603–611)	RLRAEAQVK	A3
CEF-13	Influenza	M1 (13–21)	SIIPSGPLK	A11
CEF-14	EBV	EBNA 3b (399–408)	AVFDRKSDAK	A11
CEF-15	EBV	EBNA 3b (416–424)	IVTDFSVIK	A11
CEF-16	EBV	BRLF1 (134–143)	ATIGTAMYK	A11
CEF-17	EBV	BRLF1 (28–37)	DYCNVLNKEF	A24
CEF-18	Influenza	NP (418–426)	LPFDKTTVM	B7
CEF-19	EBV	EBNA 3a (379–387)	RPPIFIRRL	B7
CEF-20	Influenza	NP (380–388)	ELRSRYWAI	B8
CEF-21	EBV	BZLF1 (190–197)	RAKFKQLL	B8
CEF-22	EBV	EBNA 3a (325–333)	FLRGRAYGL	B8
CEF-23	EBV	EBNA 3a (158–166)	QAKWRLQTL	B8
CEF-24	HCMV	pp65 (378–389)	SDEEEAIVAYTL	B18
CEF-25	Influenza	NP (383–391)	SRYWAIRTR	B27
CEF-26	Influenza	M1 (128–135)	ASCMGLIY	B27
CEF-27	EBV	EBNA 3c (258–266)	RRIYDLIEL	B27
CEF-28	EBV	EBNA 3a (458–466)	YPLHEQHGM	B35
CEF-29	HCMV	pp65 (123–131)	IPSINVHHY	B35
CEF-30	EBV	EBNA 3c (281–290)	EENLLDFVRF	B44
CEF-31	HCMV	pp65 (511–525)	EFFWDANDIY	B44
CEF-32	HCMV	pp65 (417–426)	TPRVTGGGAM	B7

Presently, the predictability of peptide determinant recognition by CD8 cells in individuals who share certain HLA alleles is largely unknown. Each CEF peptide, for example, is a *bona fide* CD8 cell determinant in individuals that express the corresponding HLA allele, but do CD8 cells in all individuals who share that HLA allele uniformly recognize that peptide, and if so, do they do it in an immune dominant fashion?

It is well established that even individual mice within a given mouse strain, in which all mice express identical *H*-*2* alleles (the *H*-*2* gene system is the murine equivalent of the human MHC system), can give rise to individual peptide recognition patterns [10, 11]. For example, if a mouse strain can mount a T cell response to peptide determinants “A”, “B”, “C”, and “D” of an antigen, all mice within the strain will respond to each of these peptides if the mice are immunized with peptides “A”, “B”, “C” or, “D”. After immunization with the antigen itself, however, some mice within the strain will mount a T cell response only to determinant “A”, other mice will respond to “B”, yet others will target “C” or “D”. In these murine models, determinant recognition is predictable in the sense that only peptides “A”, “B”, “C” or, “D” will be recognized, but determining which of the peptides will be recognized by any given individual mouse was found to be so random that the term “aleatory determinant recognition” was coined [10, 11] (*Alea* means dice in Latin). How predictable is, therefore, the recognition of *bona fide* determinants in humans? This question is central to immune monitoring, when peptides (and if using the tetramer/pentamer/dextramer or other multimer approach also MHC/HLA alleles) need to be chosen for measuring the frequencies and effector classes of in vivo induced CD8 cells.

In this study we tested on 42 healthy human donors who have been subjected to high-resolution HLA-typing in order to determine the accuracy of the CEF peptide recognition predictions. We studied whether all HLA-A*0201-positive donors who have been infected with Cytomegalo-, Epstein–barr-, and Flu virus show a CD8 cell response to the pre-defined HLA-A*0201-restricted peptides of these viruses. And, if the donor responded, we distinguished whether the response is dominant, one of several weaker (subdominant) responses, a barely detectable (cryptic) response, or whether the predicted peptide was not recognized at all, while responses to other peptides of the virus prevailed. We also established the number of times unpredicted peptides of the virus were recognized in a dominant fashion. Therefore, to the practical end, we asked, whether reliance on select “immunodominant” peptides is a reliable alternative to agnostic immune monitoring with peptide pools.

## Materials and methods

### Human subjects and peripheral blood mononuclear cells (PBMC)

Forty-two HLA-A*0201-positive healthy human donors were selected from CTL’s ePBMC library. All donors were high-resolution HLA-typed for A, B, and C alleles, and were seropositive for Cytomegalovirus (CMV), Epstein–barr virus (EBV), and Influenza virus. The subjects tested in this study were healthy adults aged 22–45. These PBMC donors were recruited by HemaCare (Van Nyus, Ca), and the PBMC were isolated by leucapheresis at HemaCare using HemaCare IRBs. The PBMC were cryopreserved at CTL (Cleveland, OH) and stored in vapor liquid nitrogen until testing in an Enzyme-linked immunospot (ELISPOT) assay. Thawing, washing, and counting of the cryopreserved cells were done as previously described [12]. Within maximally 2 h after thawing, the cells were transferred into the ELISPOT test system. In select experiments, CD4 + or CD8 + T cell subsets were depleted from PBMC using a magnetic bead selection kit (Stem Cell Technologies, Canada). The depletion assay was performed according to manufacturer’s instructions.

### Antigens

The individual CEF peptides, with amino acid sequences specified in Table 1, were purchased from Panatecs (Heilbronn, Germany) and were of more than 95 % purity. The peptides were dissolved in CTL-Test Medium (CTLT-005 –by CTL, Shaker Hts., OH, USA) and added to the ELISPOT test system at a final concentration of 1 µg/mL. HCMVpp consists of a pool of 138 15-mer peptides that cover the sequence of the HCMVpp65 protein—this peptide pool, Pepmix HCMVpp65, was purchased from JPT Peptides (Berlin, Germany).

### Human interferon-γ ELISPOT assay

The human interferon-γ (IFN-γ) ELISPOT assay was performed as we previously described [13]. Briefly, the PVDF membrane was coated with capture antibody overnight. Peptide antigens were plated first in a volume of 100 μL per well at 2× the final concentrations. The plates containing the antigen were stored at 37 °C in a humidified CO_2_ incubator until the PBMC were ready for plating. The thawed PBMC were added at 4 × 10^5^/cells per well in 100 μL using wide-bore pipette tips. Plates were gently tapped on each side to ensure even distribution of the cells as they settled and incubated for 24 h at 37 °C in a humidified CO_2_ incubator. Following completion of the ELISPOT detection, the plates were air-dried in a laminar flow hood prior to analysis. The ELISPOT plates were scanned and analyzed using an ImmunoSpot^®^ S5 Ultimate Reader from CTL. The number of spots (spot-forming units, SFU) was automatically calculated by the ImmunoSpot^®^ Software (CTL, Shaker Hts., OH) for each antigen stimulation condition versus the medium (negative) control using the SmartCount™ and Autogate™ functions [14, 15]. In all experiments and for all donors, the negative control wells had less than 10 SFU per well. Spot counts reported for the respective antigen-stimulated test conditions are means and standard deviation from triplicate wells, without the medium control subtracted.

### Statistical analysis

Mean and standard deviation from triplicate wells were calculated. Student’s *t* test was used to determine significance. Test results were defined as negative if the* p* value did not reach 0.05.

## Results and discussion

### Dominant, subdominant, and cryptic CD8 cell responses

PBMC of 42 HLA-A*0201-positive healthy human donors were tested in an IFN-γ ELISPOT assay for reactivity for each of the 32 individual CEF peptides. Operating at single-cell resolution, as performed, the IFN-γ ELISPOT assay establishes the number of peptide-triggered IFN-γ-producing cells within the 400,000 PBMC plated per well [16]. CEF peptide-induced IFN-γ production has been established by others [9] and by us [17] as CD8 cell-derived. Therefore, this assay establishes the frequency of peptide-reactive CD8 cells within the PBMC, providing a measure for the clonal size of peptide-reactive CD8 cells in a given PBMC donor, that is, the magnitude of CD8 cell immunity that targets a particular peptide.

The magnitudes of recall responses detected were divided into three categories. The “immune dominant” category was assigned to responses exceeding 100 spots per well (with 400,000 PBMC plated per well, this corresponds to a frequency of >0.04 %). One hundred spots per well represents a very strong and clear-cut response in ELISPOT as the background in these assays was for each PBMC donor less than 10 spots per well, and in many cases zero spots per well (all data not shown—examples are provided in Fig. 6b). The 0.04 % cutoff for this category is relevant with regard to data comparisons using flow cytometry-based frequency measurements of peptide-stimulated CD8 cells by staining for intracellular IFN-γ as the detection limit of Intracellular cytokine staining (ICS) is in this range, around 0.01 % [18]. Responses that were established as dominant by the more sensitive ELISPOT assay, therefore, can be expected to be detectable by ICS, albeit at the detection limit of ICS.

The detection limit for ELISPOT as performed, plating 400,000 PBMC/well, is one IFN-γ-producing cell in 400,000 cells. As ELISPOT counts among replicate wells follow a normal distribution the Student’s *t* test is suited to identify positive responses [19]. All peptides were tested in triplicate wells, and the spot counts were compared to the medium control, also measured in triplicate wells. Test results were defined as negative if the *p* value did not reach 0.05. Test results that reached this cutoff for significance but were less than 30 spots per well were defined as weakly positive, i.e., cryptic. Test results in between cryptic and dominant were called subdominant.

### CEF peptide recall responses for individual donors

**Fig. 1:**
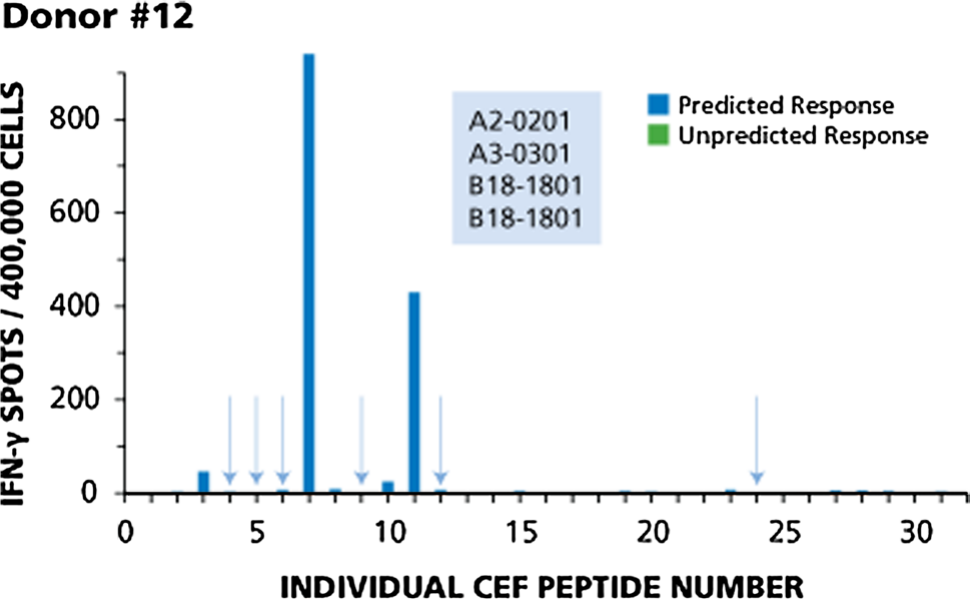
Example of immune dominance for two predicted peptides, and lack of response to six other predicted peptides (Donor # 12). The HLA type of this donor is shown in the insert. The data are described in the Text. Predicted responses are in blue and predicted responses to which this donor did not respond are highlighted by *arrows*

**Fig. 2:**
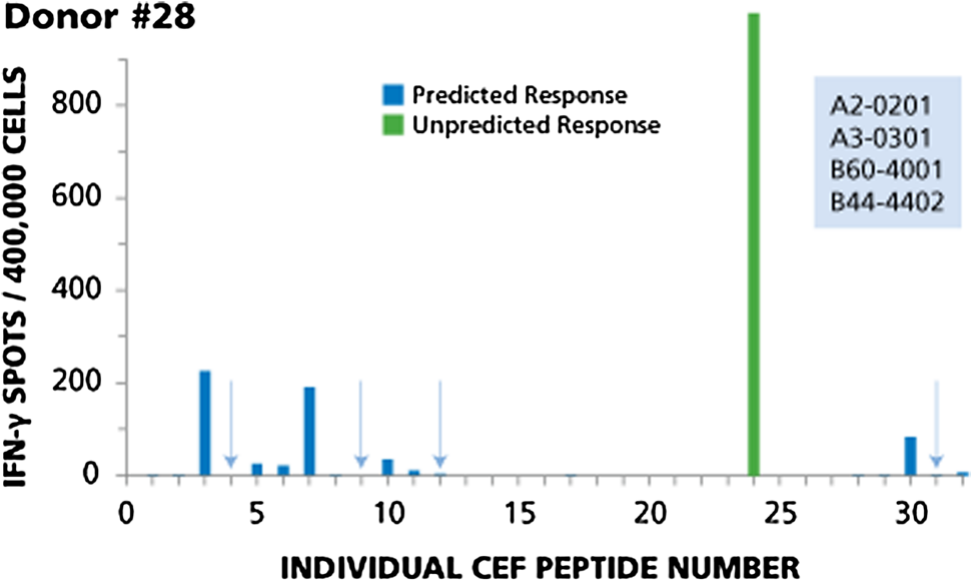
Example of immune dominance for an unpredicted peptide with magnitude of response higher than predicted dominant responses, while four predicted peptides are not recognized (Donor #28). The HLA type of this donor is specified in the insert. The results for this donor are described in the Text. Predicted responses are in *blue*, unpredicted responses in *green*. Predicted responses that scored negative are highlighted by *arrows*

**Fig. 3:**
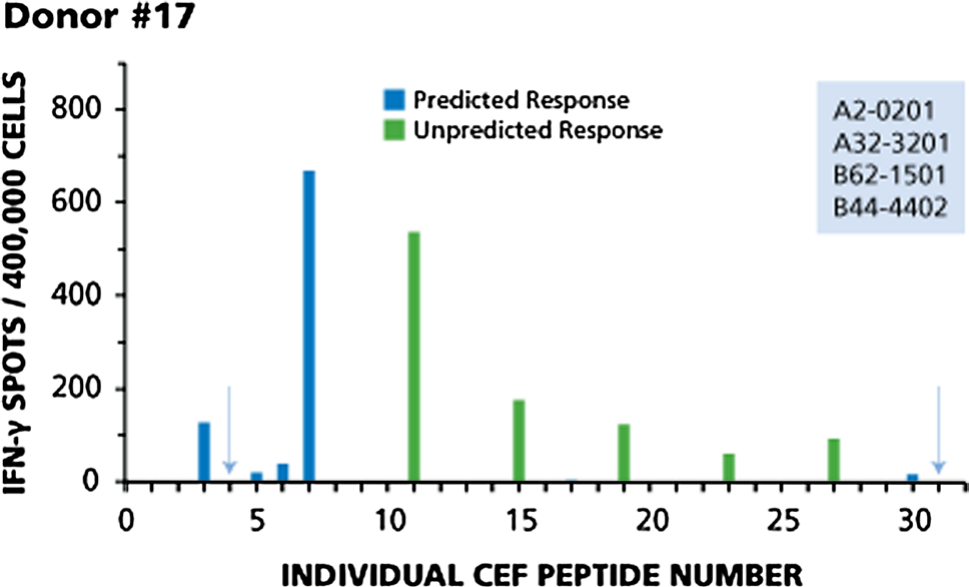
Immunodominant, subdominant and cryptic responses with both predicted and non-predicted peptides (Donor #17). Legend to Fig. 2 applies

**Fig. 4:**
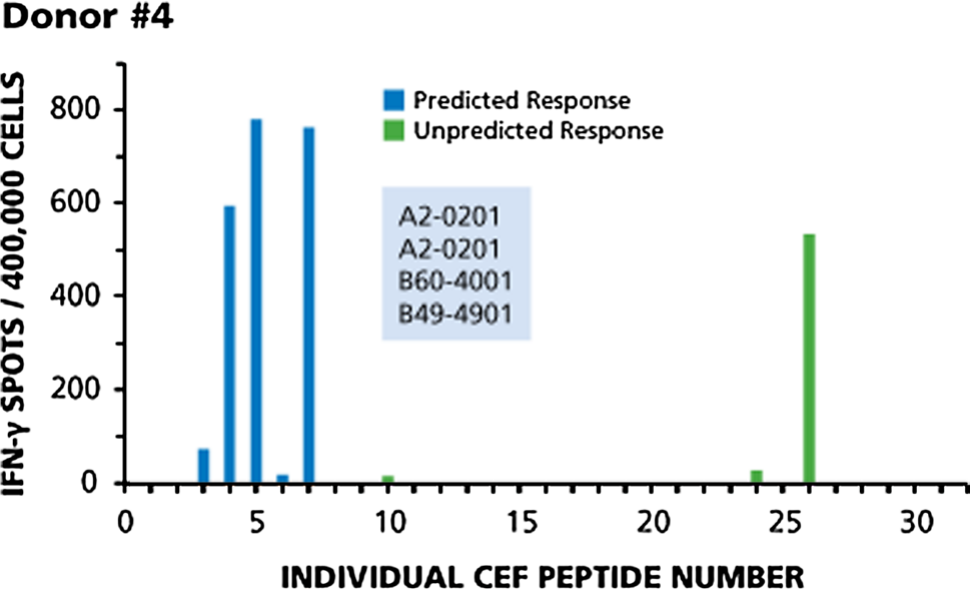
Only one donor out of the 42 tested responded to all predicted peptides, but also in a codominant fashion to an unpredicted one. Legend to Fig. 2 applies

Four donors with typical response patterns are shown in Figs. 1, 2, 3, and 4. Responses of Donor 12 are shown in Fig. 1. This donor responded to four of ten predicted peptides. Based on the HLA type of this donor (shown in the insert of Fig. 1), one would expect responses to CEF-3 and CEF-4 (A2-restricted flu peptides), CEF-5 and CEF-6 (A2-restricted EBV peptides), CEF-7 (A2-restricted CMV peptide), CEF-9 and CEF-10 (A3-restricted flu peptides), CEF-11 and CEF-12 (A3-restricted EBV peptides), and CEF-24 (a B18-restricted CMV peptide). This donor responded in a dominant fashion to two of the predicted peptides, CEF-7 and CEF-11, subdominant to CEF-3, cryptic to CEF-10, but did not respond to the six other predicted peptides (highlighted by the arrows). There was no unpredicted response in this donor.

Figure 2 shows the test results for Donor 28. This donor provides an example for immune dominance of an unpredicted peptide with magnitude of response higher than predicted dominant responses, while four predicted peptides were found to be negative. Based on the HLA type of this donor, one would expect to detect responses to 11 peptides: CEF-3 and CEF-4 (A2-restricted flu peptides), CEF-5 and CEF-6 (A2-restricted EBV peptides), CEF-7 (A2-restricted CMV peptide), CEF-9 and CEF-10 (A3-restricted flu peptides), CEF-11 and CEF-12 (A3-restricted EBV peptides), CEF-30 (B44-restricted EBV peptide), and CEF-31 (B44-restricted CMV peptide). Of these, two peptides were recognized in a dominant fashion (CEF-3 and CEF-7), two were subdominant (CEF-10 and CEF-30), and responses to the other three were cryptic (CEF-5, CEF-6, and CEF-11). The dominant response was to an unpredicted peptide, CEF 24, originally described as an HLA-B18-restricted CMV peptide, but this donor is B18 negative. CEF-24 apparently can also bind to one of the Class I alleles expressed by this donor. Four predicted peptides were not recognized: CEF- 4, CEF-9, CEF-12, and CEF-31 (highlighted by arrows).

Donor 17 (Fig. 3) provides an example of a CD8 cell response pattern in which immunodominant, subdominant, and cryptic responses encompass both predicted and non-predicted peptides. In this donor, predicted responses to seven peptides can be expected: CEF-3 and CEF-4 (A2-restricted flu peptides), CEF-5 and CEF-6 (A2-restricted EBV peptides), CEF-7 (A2-restricted CMV peptide), CEF-30 (B44-restricted EBV peptide), and CEF-31 (B44-restricted CMV peptide). This donor responded to five of the seven predicted peptides. A dominant response was seen to CEF-3 and CEF-7, a subdominant response to CEF-6, and cryptic responses to CEF-5 and CEF-30. This donor produced five unpredicted responses: dominant responses to CEF-11, CEF-15, and CEF-19, and subdominant responses to CEF-23 and CEF-27. Two predicted peptides were not recognized: CEF- 4 and CEF-31 (highlighted by arrows).

Figure 4 shows the recall response for Donor 4. This donor was the only one of the 42 tested who responded to all predicted peptides, but also in a codominant fashion to an unpredicted one. This HLA-A2 homozygotic donor responded to all five predicted A2-restricted peptides, mounting codominant responses to CEF-4, CEF-5, and CEF-7, a subdominant response to CEF-3, and a cryptic response to CEF-6. While being HLA-B27 negative, this donor showed an unpredicted codominant response to CEF-26 that was originally described as an HLA-B27-restricted peptide. This unpredicted response was of the same order of magnitude as three predicted responses, and more than 10 times stronger than the predicted responses to CEF-3 and CEF-6. Unpredicted cryptic reactivities were seen to CEF-10 and CEF-24 that were originally described as A3- and B18-restricted, respectively.

### Test results summarized for the 42 donors

**Fig. 5:**
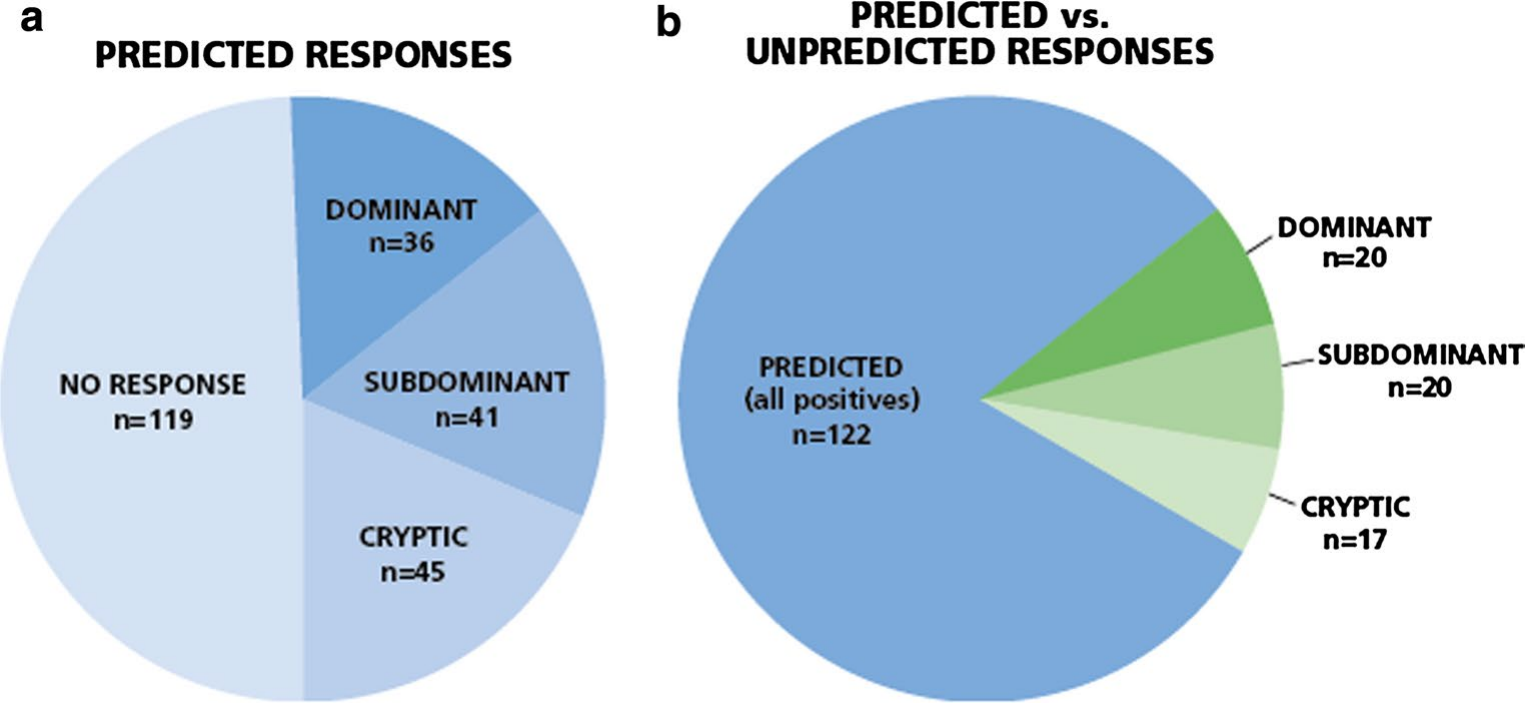
*Predicted versus actually detected responses for all donors*. Forty-two donors were tested for the 32 individual CEF peptides. **a** Of the predicted 241 recall responses, in 122 instances recall responses of various magnitudes were detected, graded as dominant, subdominant, and cryptic, as specified in the Text. In 119 instances no response was detected. **b** Predicted versus unpredicted responses among positives. Fifty-seven unpredicted responses (shown in *green*) were seen versus the 122 predicted responses. Within the unpredicted responses 20 (35 %) were dominant, 20 (35 %) were subdominant, and 17 (30 %) were cryptic

In all 42 donors, a total of 241 CEF peptide responses were predicted based on each donor’s HLA type. In 122 instances, (51 %) responses were detected, and in 119 instances (49 %) no response was detected (Fig. 5a). Thus, in approximately half of the cases the prediction was accurate, and in the other half it was not. However, within the predicted responses that actually scored positive, about one-third (45 of 122, 37 %) were weak to borderline positive, that is cryptic. In only 15 % of the predicted cases was the actual recognition of the peptide dominant (36 of 241), and in 17 % it was subdominant.

Fifty-seven unpredicted responses were seen versus the 122 predicted responses. Thus, of all the 179 responses detected, 68 % were predicted and 32 % were not. Within the unpredicted responses 20 (35 %) were dominant, 20 (35 %) were subdominant, and 17 (30 %) were cryptic (Fig. 5b). Thus, 70 % of the non-predicted responses were prevalent, that is dominant or subdominant. These non-predicted responses are likely to contribute to host defense to an equal extent as the predicted ones, and one would miss them focusing only on predicted responses.

### Weak or negative response to a single predicted peptide can misrepresent immunity to the antigen

**Fig. 6:**
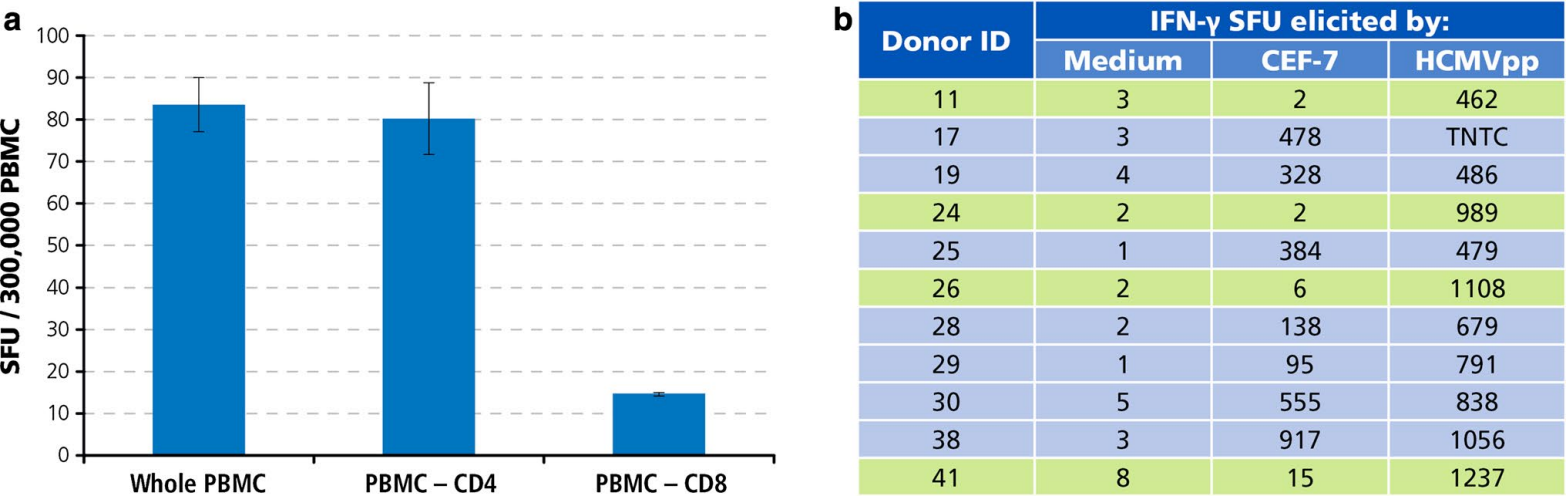
Individual donors who do not respond to an allegedly immune dominant peptide of HCMV, CEF-7, can vigorously target other determinants of the virus. **a** HCMVpp triggers IFN-γ production primarily by CD8 cells. Unseparated whole PBMC, and CD4- or CD8-cell-depleted PBMC were tested, as specified. The mean and SD of test results obtained in triplicate wells are shown. **b** Test results for HLA A2-0201-restricted CEF-7 peptide versus the HCMVpp peptide pool. CEF7 negative donors are highlighted

In several PBMC donors, CD8 cells did not target peptides that were predicted to be immune dominant. Is this because those donors did not develop strong immunity to the antigen, or because the donor responds strongly to the antigen, but to different peptides of the antigen? From the immune diagnostic perspective, this is the most critical distinction. For example, CEF-7 has been described as the immune dominant peptide of HCMV in HLA A2-positive donors [20], yet four of the 42 HCMV antibody-positive donors tested above were found to be negative for CEF-7. We therefore tested our donors for the recall response to HCMVpp, a peptide pool that consists of 138 15-mer peptides which cover the sequence of the HCMVpp65 protein. While—based on peptide length—this peptide pool is advertised to primarily activate CD4 cells, cell separation experiments using CD4 and CD8 cell depletion by magnetic beads showed that HCMVpp is prevalently recognized by CD8 cells (Fig. 6a). As shown in Fig. 6b, all four donors who have been identified as CEF-7 negative (highlighted) mounted a vigorous T cell response against HCMVpp. The magnitude of HCMVpp-reactive T cells in these CEF7-negative donors was comparable to those donors who mounted a strong CEF7 response (Fig. 6b). The frequencies of CEF-7 peptide-reactive T cells, therefore, did not reflect on the frequency of T cells that targeted other determinants of the virus. In the case of HCMV, relying on test results obtained with a single predicted peptide can provide false-negative immune diagnostic information. What about EBV and Influenza are single peptides representative of the overall anti-viral response?

Several EBV peptides are contained within the CEF pool, namely CEF 5, 6, 11, 12, 14, 15, 16, 17, 21, 22, 23, 27, 28, and 30. Taking a closer look at the individual donors, these EBV peptides seem to be recognized by CD8 cells in an apparently random (aleatory) manner. In Donor 12 (see Fig. 1), CD8 cells target EBV peptide 11 in a dominant fashion, but do not respond detectably to predicted EBV peptides 5, 6 and 12. In Donor 28 (see Fig. 2), EBV peptide 30 induces only a subdominant response and peptides 5 and 6 are cryptic, while peptides 11 and 12 are not targeted at all. This apparently aleatory pattern within the EBV recall response holds up for the remainder of the donors as well (data not shown), and it can also be seen for the Flu peptides CEF1, 2, 3, 4, 8, 9, 10, 13, 18, 20, 25, 26. These findings further confirm that it can be misleading to conclude the overall strength with which a donor responds to other determinants of the same antigen from the magnitude of the CD8 cell response to a single peptide, and thus, about the clonal sizes of T cells available in the host to combat the antigen.

## Conclusion

The data we obtained clearly show that predicted peptides are not necessarily immune dominant. In 51 % of the test cases, the predicted peptide did not induce a detectable recall response. When it did, it was one of several targeted determinants among which it was subdominant or cryptic. Thus, reliance on one or a few peptides can miss the majority of the antigen-specific CD8 cells, strongly arguing for the use of peptide pools for immune monitoring. One would predict that the larger the peptide pool, the more comprehensive the assessment of clonal sizes to an antigen is likely to be. In an ideal approach, nonamer peptides would be synthesized that walk the entire sequence of the antigen amino acid by amino acid, providing every possible determinant that can be recognized. Since ELISPOT is a high-throughput suitable assay, the peptides could be tested individually as we did previously [8, 21–27], or with peptides pooled per test condition.

Recently 384-well ELISOT plates have been introduced that permit to test one-third of the PBMC cell number per well compared to standard 96-well plates, resulting in a precisely proportional (one-third) reduction of the spot count per well over a wide range of PBMC numbers plated per well [28]. Thus, the 384-well approach lends itself to detecting dominant and subdominant peptide responses. Using the 384-well approach, as few as 30,000 PBMC per well can be tested in this way providing 30 data points with one million PBMC [28]. Thus, a typical blood draw of 20 ml would suffice to test up to 600 individual peptides or peptide pools.

When pooling peptides it is important to know the number of peptides that can be pooled without interference in the test system. We addressed this question using the very same CEF peptides, by testing the responses elicited by the individual peptides as was done in this study versus the response elicited by the pool of these 32 peptides. The number of spot-forming units (SFU) elicited by the individual peptides added up closely the SFU number elicited by the peptide pool [29]. Peptide interferences, therefore, seem to be minor with pools up to 32 peptides.

The data presented here draw attention to the need for “agnostic” testing of large peptide libraries rather than relying on immune monitoring with one or few predicted peptides.

The data presented here are based on analyzing anti-viral immunity. The T cell response to viruses follows more basic rules than immune responses to tumors. Most viruses are foreign antigens that do not affect negative and positive selection of the pre-immune T cell repertoire. In the absence of repertoire selection, it is primarily the efficacy of antigen processing and presentation that defines whether or not a peptide will become immune dominant. In the case of autoantigens and tumor antigens, the impact of these self-proteins on shaping the T cell repertoire needs to be considered. For example, immunization of myelin basic protein (MBP)-deficient mice permits to study which determinant of this antigen is immune dominant when MBP is a foreign antigen. Strikingly, this determinant is cryptic in normal mice, in which endogenous MBP has contributed to shaping the T cell repertoire [21]. By analogy, also for tumor antigens, those peptides that are predicted to be the best binders may not be immune dominant, but the opposite; they may be the ones to which the T cell system cannot respond because these peptides induced tolerance. Such considerations further complicate the observations presented here: if immune dominance cannot be reliably predicted for the relatively simple situation of the anti-viral T cell response, how could it be predicted for the more complex anti-tumor/autoimmune setting? The pragmatic conclusion to which this study leads, should apply even more for immune monitoring to cancer, however, instead of relying on select “dominant” peptides, one might be better served by using peptide pools of the antigen.

## Abbreviations

APC: Antigen-presenting cells
CD: Cluster of differentiation
CEF: Cytomegalo-, Epstein–barr-, Influenza virus
CMV: Cytomegalovirus
EBV: Epstein–barr virus
ELISPOT: Enzyme-linked immunospot assay
HLA: Human leukocyte antigen
ICS: Intracellular cytokine staining
MHC: Major histocompatibility complex
PBMC: Peripheral blood mononuclear cells
SFU: Spot-forming unit
TCR: T cell receptors
